# Knowledge of intensive care nurses’ towards prevention of ventilator-associated pneumonia in North West Ethiopia referral hospitals, 2021: A multicenter, cross-sectional study

**DOI:** 10.1016/j.amsu.2022.103895

**Published:** 2022-06-03

**Authors:** Amare Belete Getahun, Yitayeh Belsti, Mihret Getnet, Desalegn Animut Bitew, Yibeltal Yismaw Gela, Daniel Gashaneh Belay, Bewuketu Terefe, Yonas Akalu, Mengstie Diress

**Affiliations:** a Department of Anesthesia, College of Medicine & Health Sciences, University of Gondar, Gondar, Ethiopia; b Department of Human Physiology, School of Medicine, College of Medicine and Health Sciences, University of Gondar, Gondar, Ethiopia; c Department of Reproductive Health, Institute of Public Health, College of Medicine and Health Sciences, University of Gondar, Gondar, Ethiopia; d Department of Human Anatomy, School of Medicine, College of Medicine and Health Sciences, University of Gondar, Gondar, Ethiopia; e Department of Community Health Nursing, School of Nursing, College of Medicine and Health Sciences, University of Gondar, Gondar, Ethiopia; f Department of Epidemiology and Biostatistics, Institute of Public Health, College of Medicine and Health Sciences, University of Gondar, Gondar, Ethiopia

**Keywords:** Knowledge, Nurse, Intensive care unit, Ventilator-associated pneumonia, AOR, Adjusted odds ratio, CI, Confidence interval, COR, Crude odds ratio, ETT, Endotracheal tube, ICU, Intensive care unit, STATA, Statistical software for data science, VAP, Ventilator-associated pneumonia

## Abstract

**Introduction:**

Ventilator-associated pneumonia is a common nosocomial infection that occurs in critically ill patients who are on intubation and mechanical ventilation. Nurses' lack of knowledge may be a barrier to adherence to evidence-based guidelines for preventing ventilator-associated pneumonia. This study aimed to assess the knowledge of intensive care nurses’ towards the prevention of ventilator-associated pneumonia.

**Methods:**

A multicenter cross-sectional study was conducted among nurses working in the intensive care unit from April to July 2021. A pre-tested and structured questionnaire was used to collect data. All intensive care nurses working in the study area were included in the study. Data was entered into Epi-data 4.1 version (EpiData Association, Denmark) and transferred to STATA version 14 (College Station, Texas 77845-4512 USA) statistical software for analysis. Both bi-variable and multivariable binary logistic regression analysis was used to identify factors associated with knowledge of intensive care unit nurse. Variables with a p-value less than <0.2 in the bi-variable analysis were fitted into the multivariable logistic regression analysis. Both Crude and Adjusted Odds Ratio with the corresponding 95% Confidence Interval was calculated to show the strength of association. In multivariable analysis, variables with a p-value of <0.05 were considered statistically significant.

**Result:**

A total of 213 intensive care nurses were included in the study, with a response rate of 204(95.77%). The mean knowledge score of intensive care nurses regarding the prevention of ventilator-associated pneumonia out of 20 questions is (10.1 ± 2.41). There are 98 (48.04%) of the participants have been found to have good knowledge and 106 (51.96%) of them are rendered poor knowledge about the overall knowledge related to the prevention of ventilator-associated pneumonia. Higher academic qualifications and taking intensive care unit training were significantly associated with good knowledge of ventilator-associated pneumonia prevention in multi-variable logistic regression.

**Conclusion:**

Our study indicates that the knowledge of intensive care nurses about ventilator-associated pneumonia prevention is not sufficient. Higher academic qualifications and taking intensive care unit training are significantly associated with a good level of knowledge. Therefore it shows the necessity for thorough training and education.

## Highlights

• The knowledge of intensive care nurses about ventilator-associated pneumonia prevention is not sufficient.
• Only 98 (48.04%) of the participants have been found to have good knowledge.
• Higher academic qualifications and taking intensive care unit training are significantly associated with a good level of knowledge.
• This study shows the necessity of in-service training and continuing education for intensive care nurses about the prevention of ventilator-associated pneumonia.

## 1 Introduction

Ventilator-associated pneumonia is defined as pneumonia that occurs 48–72 h or thereafter following endotracheal intubation [1]. It is the most serious healthcare-associated infection in intensive care units (ICUs) for patients undergoing mechanical ventilation [2].

Ventilator-associated pneumonia (VAP) is a common nosocomial complication arising in the ICU, it accounts for 47% of infections in patients in intensive care units [3]. In developing countries, multiple studies have found a significant increase in the incidence of VAP. In India, Costa Rica, and Iran the incidence of VAP is 40.1, 44.3, and 21.6 infections per 1,000 days of ventilation respectively [[4], [5], [6]]. Multiple factors contribute to the increasing rate of VAP such as length of stay in ICU (prolonged ventilation), older age (≥60 years), emergency intubation, coma, re-intubation, tracheostomy, prior antibiotic therapy, bronchoscopy, transfusion, transport out of the ICU[6,7].

VAP is a serious healthcare-acquired complication of mechanical ventilation associated with considerable morbidity, which can prolong the mechanical ventilation period, increase intensive care and hospital length of stay, and increased costs of hospitalization (VAP cost is reported to be as high as $10 billion per year), increase the use of antimicrobials and increase mortality risk in an intensive care unit [[8], [9], [10]].

Even though VAP is a serious problem across the world its high incidences in developing countries may be contributed by inadequate knowledge and awareness about the problem. Several strategies have been created in an attempt to find a solution to the problem of VAP in the world; these strategies incorporate many evidence-based strategies proved in the literature to decrease VAP and increase positive patient outcomes [11].

The nurses working in the ICU are in the best position to put the evidence-based knowledge into practice as they are at the patient's bedside 24 h a day and therefore they play an important role in the prevention of VAP [12]. Determining critical care nurses' knowledge of VAP prevention may be useful in improving their awareness, thus leading to better practices and preventing this serious problem in Ethiopia. Thus, this study aimed to assess the knowledge of VAP prevention among critical care nurses.

## 2 Methods

### 2.1 Study setting and population

A multi-center institutional-based cross-sectional study was conducted among nurses working in the intensive care unit at Amhara Regional State Referral Hospitals, Northwest Ethiopia, from April to July 2021. Currently, in Amhara Regional state there are six referral hospitals (University of Gondar Compressive Specialized Hospital, Tibebe Ghion Compressive Specialized Hospital, Felege Hiwot Referral Hospital, Debre Berhan Referral Hospital, Dessie Referral Hospital, and Debre Markos Referral Hospital) in which intensive care is provided for critically ill patients. Nurses who had working experience of less than 6 months, head nurses, and nurses on annual leave during data collection were excluded from the study.

Currently, there are a small number of personnel in each referral hospital in the Amhara regional state, Ethiopia. Hence, studying all the source populations is feasible in terms of budget and time and therefore, the size of the study population was the size of the source population. Hence, all nurses (N = 213) working in ICUs in those hospitals during the data collection period were invited to participate in the study for the power of representativeness through the convenience sampling technique.

The article has been registered with UIN 7699 in the research registry. The hyperlink to our specific registration is here (that can be publicly accessible and checked): https://www.researchregistry.com/browsetheregistry#home/registrationdetails/625b18315b05bb001fa3c20c/.This work has been reported in line with the STROCSS criteria [13].

### 2.2 Operational definitions

Knowledge- In this study, it refers to the correct question of response of the subject regarding the prevention of ventilator-associated pneumonia [14].

Good knowledge: Refers to those study participants who score points more than the mean of knowledge questions correctly.

Poor knowledge: Refers to those study participants who score less than or equal mean point knowledge questions correctly.

### 2.3 Data collection procedure and technique

Data were collected using a structured self-administered questionnaire regarding the knowledge of nurses about the prevention of ventilator-associated pneumonia working in the intensive care units. The head nurse from each of the ICUs will collect the data and supervise the data collection. The questionnaires comprised two parts: personal and professional characteristics and the level of knowledge of nurses regarding the VAP prevention questionnaire. Data were collected by five nurses who were the heads of each hospital's ICU who distributed the questionnaires to the respondents by getting their willingness and collected the filled data.

The first part is a socio-demographic characteristic of the nurses which included age, gender, qualification level, and years of experience, type of ICU working, and taking training on ICU infection prevention.

Part two consists of 20 questions used to assess nurses' level of knowledge, which were adopted from a reliable questionnaire developed by Vandijick [15] and from the same research done in Tanzania [16] and South Africa [17] but, modified according to the Ethiopian context. Both adopted questions were again checked and assessed for their validity with the ICU experts and others were developed and added to the questionnaire for this study.

Finally, an English version of the self-administered questionnaire was used to collect data from the nurse professionals, as the medium of instruction in all study areas of the institution is English.

### 2.4 Data quality assurance

To ensure the quality of data, pre-testing of the data collection tool (the questionnaire) was conducted on nurse professionals from an ICU center and pre-tested participant was not included in the main study and the collected data was checked for completeness, accuracy, and clarity. Then necessary corrections was made accordingly to the questionnaire for the main study. During the data collection process, each questionnaire was checked daily by the supervisor for its completeness and accuracy.

### 2.5 Data analysis and interpretation

After data collection, the data were coded, entered, and cleaned before statistical tests. Epi-data 4.1 version (EpiData Association, Denmark) was used to enter the data, and then transferred to STATA version 14 (College Station, Texas 77845-4512 USA) statistical software for analysis. Descriptive statistics were used to summarize the socio-demographic characteristics of the participants and their knowledge scores. Both bi-variable and multivariable binary logistic regression analyses were used to identify factors associated with the knowledge level of ICU nurses. Variables with a p-value less than <0.2 in the bi-variable analysis were fitted into the multivariable logistic regression analysis. Both Crude Odds Ratio (COR) and Adjusted Odds Ratio (AOR) with the corresponding 95% Confidence Interval was calculated to show the strength of association. In multivariable analysis, variables with a p-value of <0.05 will be considered statistically significant.

### 2.6 Ethical consideration

Ethical clearance was obtained from the University of Gondar College of Medicine and Health Science, School of Medicine Ethical Review Committee and an official permission letter was also obtained to conduct the research from each ICU of hospital administrator. Informed consent was obtained from each study subject after a clear explanation of the objective and purpose of the study. Confidentiality issue was ensured by avoiding personal identification on the questionnaire and by keeping the questionnaire locked.

## 3 Result

### 3.1 Socio-demographic characteristics

A total of 213 nurses working in ICUs of North West Amhara regional state hospitals were included in the study, with a response rate of 204(95.77%). One hundred twenty-seven (62.25%) of the participants were males and around half of the study participants were in the age group of 30–40 years. Nearly two-thirds of the participants (66.18%) were married. Similarly, around two-thirds of the participant had a bachelor's degree in their academic qualification. The majority of them (69.6%) had less than two years of working experience in the ICU. Around three-fourths (68.14%) of the nurses were not took formal ICU training (Table1).

**Table 1:** Socio-demographic characteristics of the study participant in Northwest Ethiopia, 2021. (n = 204).

Variable	Category	Frequency(n)	Percentage (%)
Age in years	< 30	86	42.16
	30–40	94	46.08
	> 40	24	11.76
Gender	Male	127	62.25
	Female	77	37.75
Marital status	Married	135	66.18
	Divorced	11	5.39
	Single	58	28.43
Academic qualification	Bachelor degree	134	65.69
	Master's degree	70	34.31
ICU working	Medical ICU	86	42.16
	Surgical ICU	65	31.86
	Mixed ICU	53	25.98
Year of experience	6month -2years	142	69.61
	3–6 years	49	24.02
	7–10years	6	2.94
	Above 10 years	7	3.43
Taking ICU training	Yes	65	31.86
	No	139	68.14

### 3.2 Knowledge of nurse's towards the prevention of VAP

In this study, 75.98% of respondents recognized the importance of maintaining a high nurse-to-patient ratio in the ICU for the prevention of VAP. Of respondents, 71.08% knew the importance of wearing sterile gloves during suctioning of the endotracheal tube of the ventilated patient in ICUs for prevention of VAP and almost 51% reported preferring the use of kinetic beds to reduce the risk of VAP although not all beds found in the study area are kinetic (Table2).

**Table 2:** Nurses’ answers to questions about knowledge of prevention of VAP in Northwest Ethiopia, 2021.(n = 204).

Variable	Response	Frequency(n)	Percentage (%)
Recommended Endotracheal route of intubation is?---- (Oral intubation)	Correct	94	46.08
	Incorrect	110	53.92
Recommended type of positioning for ventilated Patient (If there is no contraindication)? ------(Semi recumbent)	Correct	85	41.67
	Incorrect	119	58.33
Advantage of Endotracheal tubes with an extra lumen for drainage of subglottic secretions?----(Reducing the risk for VAP)	Correct	86	42.16
	Incorrect	118	57.84
Factors contributing to bacterial colonization of the aero-digestive tract include?-----(Contaminated hands of health workers, contaminated respiratory therapy equipment, Aspiration of secretion)	Correct	93	45.59
	Incorrect	111	54.41
A nurse is required to discard a suction catheter? (Immediately after a single use)	Correct	76	37.25
	Incorrect	128	62.75
The recommended type of suction systems for intubated patients? (Closed suctions systems)	Correct	73	35.78
	Incorrect	131	64.22
Suction catheter Insertion into the Endo-tracheal tube? (It is a sterile procedure)	Correct	86	42.16
	Incorrect	118	57.84
Head of the bed elevation should be range from? (30–45°)	Correct	86	42.16
	Incorrect	118	57.84
A nurse caring for a ventilated patient is required to wear sterile gloves during? (ETT suctioning)	Correct	145	71.08
	Incorrect	59	28.92
A nurse caring for a ventilated patient is required to wash hands? (Before and after oral/ETT suctioning)	Correct	70	34.31
	Incorrect	134	65.59
Perform oral care by using a swab moistened with mouth wash and water is recommended?--(every 4–6 h)	Correct	60	29.41
	Incorrect	144	70.59
Prolonged use of Stress ulcer prophylaxis to a ventilated patient?---(May increase the colonization density of the aerodigestive tract)	Correct	73	35.78
	Incorrect	131	64.22
Maintenance of a high nurse to patient ratio in the ICU is associated with?---(Decreased risk for VAP)	Correct	155	75.98
	Incorrect	49	24.02
Chest physiotherapy is recommended for ICU patients (to reduce the risk for VAP)	Correct	87	42.65
	Incorrect	117	57.35
If you compare the Adjustable beds with the non-adjustable ones?---(Adjustable beds reduce the risk of VAP)	Correct	104	50.98
	Incorrect	100	49.02
Frequency of ETT suctioning should be done to the patient? (As needed)	Correct	83	40.69
	Incorrect	121	59.31
Early weaning of mechanical ventilator mode results?---(Reduces the risk for VAP)	Correct	76	37.25
	Incorrect	128	62.75
Overfeeding a ventilated patient may increase the risk of aspiration leading to?--(Increased the risk for VAP)	Correct	70	34.31
	Incorrect	134	65.69
ETT tube with well-maintained pressure cuff for ventilator patient helps to? (decrease the risk for VAP)	Correct	90	44.12
	Incorrect	114	55.88
Unplanned extubation can increase the risk of aspiration leading to? (Increased the risk for VAP)	Correct	68	33.33
	Incorrect	136	66.67

In the current study, the overall mean knowledge score of ICU nurses regarding the prevention of VAP out of 20 questions is (10.1 ± 2.41). By using the mean score as a cut of value, we divide the respondents into two groups for analysis purposes, which include respondents with good knowledge (who score ≥10.1 correct answers of 20 items) and inadequate knowledge (who score <10.1 correct answers of 20 items). There are 98 (48.04%) of the participants have been found to have good knowledge and 106 (51.96%) of them are rendered poor knowledge about the overall knowledge related to the prevention of VAP.

### 3.3 Factors associated with knowledge of ICU nurses

In the bi-variable logistic regression age, academic qualification, year of experience, and taking ICU training were positively associated with good knowledge about VAP prevention. But, finally, academic qualification and ICU training were significantly associated with good knowledge of VAP prevention in multi-variable logistic regression (Table3).

**Table 3:** Bi-Variable and Multivariable Binary Logistic Regression Analyses Results of Associated Factors of knowledge of ICU Nurses, Northwest Ethiopia, 2021.

Variables	Knowledge		OR(95%CI)	
	Good n (%)	Poor n (%)	COR(95%CI)	AOR(95%CI)
Age				
≤30	30(38)	49(62)		
31-39	37(45.1)	45(54.9)	0.29(-0.33,0.92)	0.47(0.24,1.33)
≥40	31(72.1)	12(27.9)	1.43(0.63,2.24)	1.53(0.52,1.52)
Year of experience				
6month -2years	29(39.7)	44(60.3)		1
3–6 years	21(45.6)	25(54.4)	0.24(-0.5,0.98)	0.48(1.18,1.25)
7–10 years	12(37.5)	20(62.5)	0.09(-0.94,0.96)	0.57(0.19,1.64)
Above 10 years	36(67.9)	17(32.1)	1.16(0.42,1.9)	1.67(0.7,4.02)
Academic qualification				
Diploma	2	7		
Bachelor degree	23(18.6)	72(81.4)	0.12(-1.52,1.75)	1.01(0.18,4.02)
Masters degree	73(74.8)	27(25.2)	2.24(0.61,3.87)	7.45(1.38,40.26)**
TakingVAP prevention training				
Yes	45(69.2)	20(30.8)	1.29(0.66,1.92)	3.13(1.37,7.13)**
No	53(39.8)	80(60.2)		

Note: ** significant variables from multivariable regression.

## 4 Discussions

This study aimed at determining the knowledge and associated factors of VAP prevention among ICU nurses' in a resource-limited country. The mean knowledge score was 10.1 out of 20.

In this study, around 52% of the participants have a knowledge score below the mean (inadequate knowledge). This result consistent with recent studies reported in Addis Ababa [18], in Egypt [19], in Iran [20], in Tanzania [21], in Iraq [22],in Pakistan [23],in Yemen [24], in Malaysia [25] and in Taiwan [26]. However, our result showed a considerably low mean knowledge score as compared to other studies, reported in the United State of America [27], in Jordan [28]. This variation in knowledge scores in the current study can be explained by differences in healthcare delivery systems [27] and, variations in specific guidelines and policy regarding training and practice of evidence-based guidelines for VAP prevention in ICUs.

Sufficient nursing knowledge helps to give optimal patient care, establish confidence in making better decisions, and improve the outcomes of ventilated patients [29]. Developing a specific guideline and strategy for teaching VAP prevention that takes into account the problems in resource-limited settings while maintaining VAP prevention efficacy might be beneficial in decreasing knowledge gaps in resource-limited settings.

The reason for inadequate knowledge in ICU nurses is multifactorial. In the current study having high academic qualifications (Masters' degree) and taking regular VAP, prevention training was significantly associated with adequate knowledge in multivariable logistic regression. The result of our study showed that nurses having a master's degree were higher odds of adequate knowledge than nurses having a diploma or bachelor's degree. This is in agreement with the findings from previous literature [[29], [30], [31]]. The availability of a high ICU professional qualification contributes significantly to better VAP prevention.

The result of this study revealed that ICU nurses who had taken training on VAP prevention were higher odds of adequate knowledge than nurses who had not taken regular training. Which is congruence with other studies [28,32,33]. These findings were also supported by another study that revealed that continuous education and training improved both knowledge and adherence related to VAP preventive measures and significant practical improvements were observed after education sessions [29]. Continuing education opportunities for in-service nurses are uncommon in resource-constrained countries like Ethiopia; however, continuing education programs are essential to improve nurses’ knowledge of VAP prevention, nursing administrators and hospitals should use a systematic strategic and educational strategy for VAP prevention.

Unlike several previous studies [30,[34], [35], [36], [37]], our study revealed that there were no differences in VAP prevention knowledge between more experienced and less experienced nurses. These findings are in agreement with research from Iran [38], Tanzania [21], New Zealand [39], and the United States [27].

The level of knowledge regarding VAP prevention, in comparison to previous studies, in the current study seems inadequate. Although having knowledge about the principles of evidence-based care cannot ensure the implementation of these principles, a lack of knowledge might be a potential barrier to applying the evidence-based guidelines for the prevention of VAP. Guidelines for VAP prevention might change over time. Each time new evidence-based strategies for reducing VAP are developed, the questionnaire will need to be adapted and reevaluated.

The finding of this study had varies implications. In raising the average level of overall knowledge of nurse the first step is developing successful diverse educational programs. The relevance of nursing expertise and an evidence-based practice approach to VAP prevention is emphasized in the literature. This study is useful because it will generate questions and hypotheses for future studies.

## 5 Limitations of the study

As a limitation, primarily this study is institutional-based which could limit its generalizability and the cross-sectional nature of the study would also limit its ability to establish a temporal relationship. Second, since we are used a convenience sample which may not provide a representative result. Third, we have been unable to identify the effect of the work environment on VAP knowledge prevention among critical care nurses.

## 6 Conclusion and recommendations

Our study indicates that the knowledge of intensive care nurses about VAP prevention is not sufficient. Higher academic qualifications and taking intensive care unit training are significantly associated with a good level of knowledge. Therefore it shows the necessity for thorough training and education. The school of nursing should revise the nursing curriculum in Ethiopia and incorporate intensive care infection prevention initiatives. In addition policymakers and administrators should give attention to implementing and updating VAP prevention guidelines, which would be useful for improving the quality of nursing care and increasing the knowledge of the nurses to make the right decisions.

## Ethical approval

This study was approved by the Ethics Committee of the University of Gondar Comprehensive Specialized Hospital and was performed in accordance with the Helsinki Declaration of 1964 and later amendments. Informed written consent was obtained from each study subject after a clear explanation of the objectives and purposes of the study. Participants were informed of their right to refuse to participate in the study at any time. Confidentiality was ensured by avoiding personal identification on questionnaires and by keeping the questionnaires locked.

## Sources of funding

No funding was received.

## Author contribution

This work was carried out in collaboration among all authors. ABG, YB, MG, and DAB participated in data entry, analysis, interpretation, manuscript preparation, and providing the final version of the study. YYG, DGB, and BT participated in the conception of the design, proposal writing, and edition, contributed to the interpretation of the results, and revised the paper at the final version. YA, MD contributed in edition of the final version and contributed in analysis and interpretation. All authors read and approved the submitted manuscript.

## Consent

Participants were well informed and agreed with no benefit was obtained.

## Registration of research studies

1. Name of the registry: Research registry.

2. Unique Identifying number or registration ID: 7815.

3. Hyperlink to your specific registration (must be publicly accessible and will be checked):https://www.researchregistry.com/browsetheregistry#home/registrationdetails/625b18315b05bb001fa3c20c/

## Guarantor

Amare Belete Getahun (ABG), Yitayeh Belsti (YB), Mihret Getnet (MG), Desalegn Animut Bitew (DAB), Yibeltal Yismaw Gela (YYG), Daniel Gashaneh Belay (DGB), Bewuketu Terefe (BT), Yonas Akalu (YA), Mengstie Diress (MD).

## Provenance and peer review

Not commissioned, externally peer-reviewed.

## Declaration of competing interest

There is no conflict of interest.
